# The Effect of Altered Loading on Mandibular Condylar Cartilage

**DOI:** 10.1371/journal.pone.0160121

**Published:** 2016-07-29

**Authors:** Raman Kaul, Mara H. O’Brien, Eliane Dutra, Alexandro Lima, Achint Utreja, Sumit Yadav

**Affiliations:** 1 Division of Orthodontics, University of Connecticut Health Center, Farmington, United States of America; 2 Division of Orthodontics, Indiana University Purdue University Indianapolis, Indianapolis, United States of America; University of Pittsburgh, UNITED STATES

## Abstract

**Objective:**

The purpose of this study was to delineate the cellular, mechanical and morphometric effects of altered loading on the mandibular condylar cartilage (MCC) and subchondral bone. We hypothesized that altered loading will induce differentiation of cells by accelerating the lineage progression of the MCC.

**Materials and Methods:**

Four-week-old male Dkk3 XCol2A1XCol10A1 mice were randomly divided into two groups: (1) Loaded-Altered loading of MCC was induced by forced mouth opening using a custom-made spring; (2) Control-served as an unloaded group. Mice were euthanized and flow cytometery based cell analysis, micro-CT, gene expression analysis, histology and morphometric measurements were done to assess the response.

**Results:**

Our flow cytometery data showed that altered loading resulted in a significant increase in a number of *Col2a1*-positive (blue) and *Col10a1*-positive (red) expressing cells. The gene expression analysis showed significant increase in expression of *BMP2*, *Col10a1* and *Sox 9* in the altered loading group. There was a significant increase in the bone volume fraction and trabecular thickness, but a decrease in the trabecular spacing of the subchondral bone with the altered loading. Morphometric measurements revealed increased mandibular length, increased condylar length and increased cartilage width with altered loading. Our histology showed increased mineralization/calcification of the MCC with 5 days of loading. An unexpected observation was an increase in expression of tartrate resistant acid phosphatase activity in the fibrocartilaginous region with loading.

**Conclusion:**

Altered loading leads to mineralization of fibrocartilage and drives the lineage towards differentiation/maturation.

## Introduction

The temporomandibular joint (TMJ) is a complex load bearing joint in the craniofacial complex. Numerous investigators have shown that the TMJ is a load bearing joint and the uniqueness of the mandibular condylar cartilage (MCC) lies in its ability to be remodeled by mechanical loading[1–3]. While reduced loading and overloading causes cartilage degradation, a moderate level of activity is necessary for maintenance of cartilage integrity and joint homeostasis. Mechanical stimulation of the MCC has been shown to generate biochemical signals, which increases the anabolic activity of chondrocytes[4, 5].

The MCC is composed of four different zones: the fibrous, proliferative, pre-hypertrophic and hypertrophic[6–8]. The proliferative zone separates the fibrocartilaginous part of MCC with the more mature hyaline cartilage (pre-hypertrophic and hypertrophic zones of the MCC)[8]. The MCC is a load bearing musculoskeletal tissue, which distributes stress [9, 10]. The nature and duration of applied loads determine the biomechanical properties of the MCC. Small changes in the integrity, composition, or organization of cellular components of the cartilage will alter the matrix production and may eventually alter its mechanical properties. The literature lacks cellular details regarding altered loading of the MCC and none of the published studies have clearly elucidated the cellular morphometry and associated changes in the morphology of the cartilage and associated subchondral bone.

The *Dickkopf* family of genes encodes secreted proteins that primarily act as antagonists of the Wnt/β-catenin signaling pathway. Of the four main *Dkk* family members in vertebrates (Dkk 1,2,3,4), *Dkk3* differs from the rest, both structurally and in chromosomal location, indicating a functional divergence into two *Dkk* subfamilies[11–13]. The role of *Dkk3*, specifically in cartilage development, has not been investigated; however, upregulation of the gene was observed during pathological states. A comparison of gene expression profiles between normal and osteoarthritic cartilage demonstrated consistently upregulated *Dkk3* levels in osteoarthritis, suggesting its involvement in disease progression[14]. Addressing the question of whether *Dkk3* has a pro- or anti-inflammatory effect in this situation, it was recently shown that *Dkk3* inhibits matrix degradation by inflammatory cytokines in osteoarthritis, thus protecting the cartilage.

Our study employs a combination of strategies (Fluorescence activated cell sorting analyses, micro-CT, histology, morphometric measurements and gene expression) to study the effect of altered loading on the MCC. The primary objective is to study the effects of mechanotransduction at the cellular level after altered mechanical loading. The objectives were achieved by determining the spatial and temporal changes in Dkk3-eGFP, Col2a1-CFP, and Col10a1-RFP transgene expression, tissue remodeling (tartrate resistance acid phosphatase, TRAP), enzymatic indicator of mineralization (alkaline phosphatase, AP) and cartilage proteoglycan distribution (toluidine blue staining) after altered loading. We believe understanding the cellular changes due to altered mechanical loading of the MCC may contribute to our understanding of the mechanism underlying condylar growth modification in response orthodontic forces. In this research we utilized the forced mouth-opening model published by Sobue et al with minor modifications [5]. Our objectives were to study the 1) the cellular changes in the MCC with the altered loading; 2) tissue level changes in the subchondral bone and mineralized cartilage. Our null hypothesis is that there is no difference in cellular (cell proliferation and cell types), structural and morphometric characterization in the MCC and subchondral bone in the altered loaded model as compared to the healthy animals (control group).

## Materials and Methods

### GFP reporter mice

The Institutional Animal Care Committee of the University of Connecticut Health Center approved this animal study. We used 4 week old (postnatally), male, triple transgenic mice (Dkk3 X Col2a1 X Col10a1) on a CD-1 background for the study. The Dkk3-eGFP transgene was obtained from the MMRRC repository (MMRRC: MGI: 4846992) (http://www.mmrrc.org). Dkk3-eGFP transgene was developed from a bacterial artificial chromosome (BAC) containing eGFP in the first exon of the murine Dkk3 gene. The two other GFP transgenes (Col2a1-GFPcyan and Col10a1-RFPcherry) used in this study has been previously described[15, 16]. All three GFP reporters were bred to make triple transgenic mice used in this study[17]. After the experimental procedure animals were killed with CO_2_ asphyxiation; the MCC along with sub-chondral bone was quickly dissected free by cutting the muscular attachment without scrapping the cartilage of the condyle.

### Experimental Procedure

**Fig 1:**
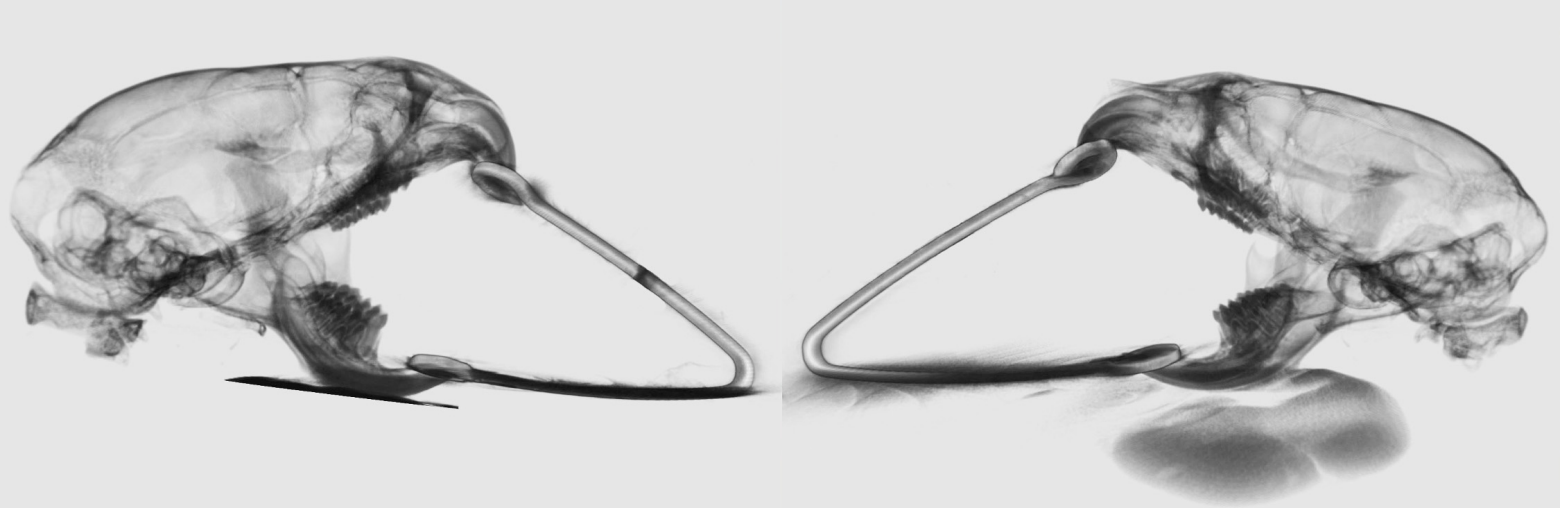
Altered loading due to forced mouth opening. The compressive force on the mandibular condylar cartilage due to forced mouth opening. The loading of the MCC is symmetrical between the right and the left side of the TMJ.

**Fig 2:**
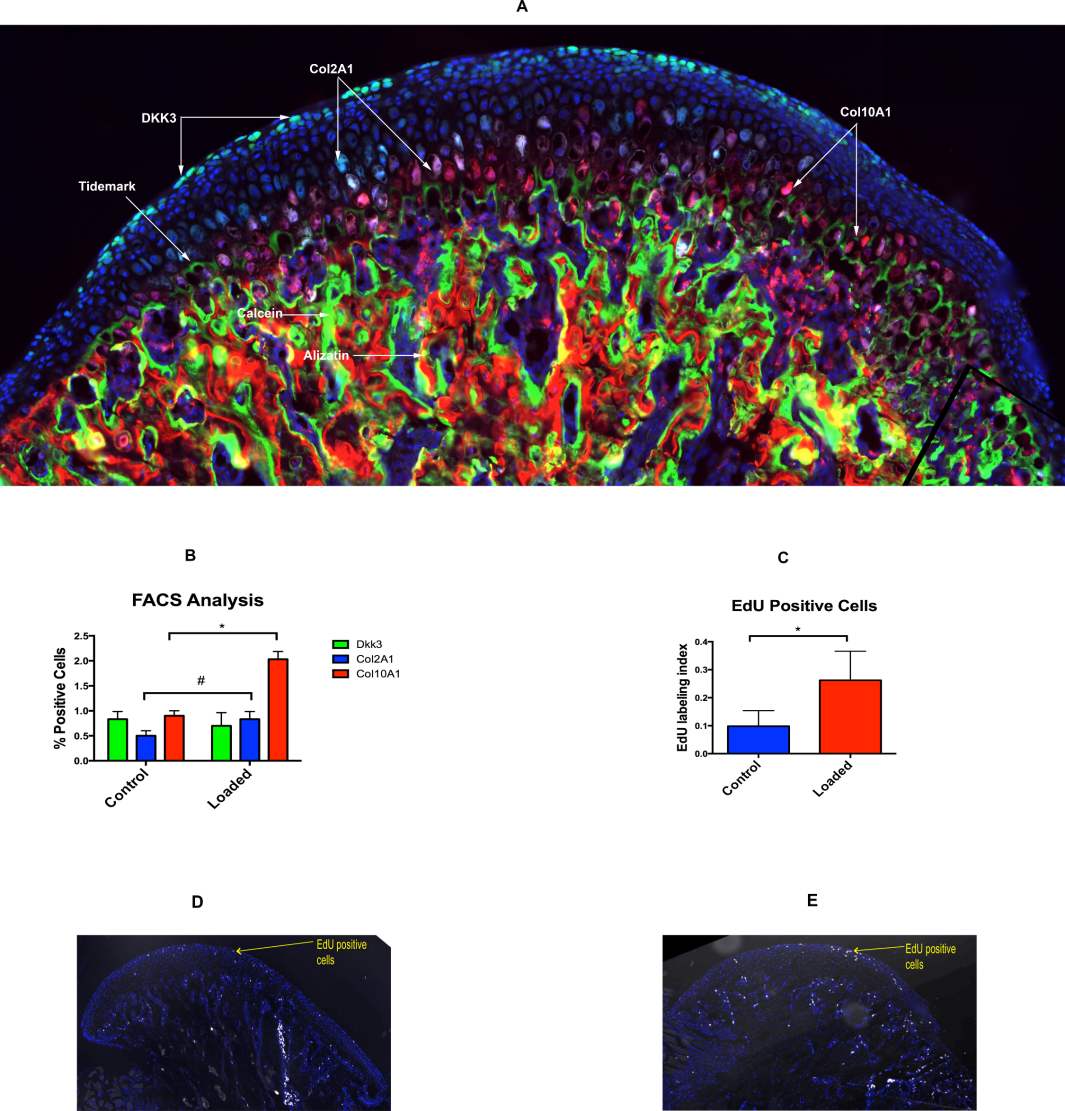
Altered loading leads to increased proliferation and differentiation. (2A): The histological section of mandibular condylar cartilage from a 4 week-old-mice (Dkk3xCol2a1xCol10a1). Dkk3-positive (green) cells are present in the superficial zone/ fibrocartilaginous zone of MCC, Col2A1-positive (blue) cells are present in the pre-hypertrophic region of mature cartilage and Col10A1-positive (red) cells are present in the hypertrophic zone, adjacent to tidemark. Distinct bone labels in the subchondral bone. (2B): Flow cytometry based cell analysis (percentage of total positive cells) (*: p<0.05 = significant difference between the altered loading and unloaded (control) group. (2C): EdU labeled proliferating cells were significantly higher in the loaded group when compared to control group (*: p<0.05 = significant difference between the altered loading and control group). (2D): Sagittal section of MCC in the control group showing EdU positive cells in the superficial layer and proliferating layer. (2E): Sagittal section of MCC in the altered loaded group showing more number of EdU positive cells in the superficial layer and proliferating layer. Histograms represent means ± SD. Scale bar = 500μm.

The mice were divided into 2 groups: (1) Experimental Group (n = 15): Altered loading through forced mouth opening continuous for 1hr/day for 5 days (Fig 1); (2) Control Group (n = 15): Unloaded group with incisor trimming (mandibular) every alternate day (Table 1). The mice in experimental and control groups were anesthetized with a mixture of ketamine (90mg/kg) and Xylazine (13mg/kg). In the experimental group, the mice were loaded with the custom made springs as described by Sobue et al[5]. However, our springs were made from Connecticut New Archform (CNA) wire (0.32inch) and applied 0.5N of force as measure by the Correx tension gauge (Haag-Streit, Bern, Switzerland). All the mice were injected with alizarin (3μg/kg body weight) on the third day and calcein (3μg/kg body weight) on the fifth day intraperitoneally. Further they were injected with 5-ethnyl-2’-deoxyuridine (30mg/kg body weight) intraperitoneally, 24 hours before euthanization. The mice in the experimental and control groups were euthanized 24 hours after the force application on the 5^th^ day.

**Table 1:** Number of Animals.

Mouse	Tota(n)	Age(weeks)	FlowActivatedCellSorting (n)	Real-time PCR(n)	Micro CT(n)	Histolog(n)
Experimental(Dkk3xCol2a1xCol10a1)	15	3–4	9condyles	9condyles	12condyles	12condyles
Control(Dkk3xCol2a1xCol10a1)	15	3–4	9condyles	9condyles	12condyles	12condyles

### Histomorphometry

The MCC of the TMJ along with the sub-chondral bone were fixed for 24hours in 10% formalin and were placed in 30% sucrose overnight and embedded in cryomedium (Thermo Shandon, Pittsburgh, PA) using disposable base molds (Thermo Shandon, Pittsburgh, PA). The medial surfaces of the samples were embedded against the base of the mold and were parallel to the floor of the mold. Specimens were stored at -20°C or -80°C before they were sectioned using Leica cryostat (Nussloch, Germany). Sections were 5 to 7μm in thickness and were transferred to slides using a tape transfer method[18]. Sequential sections were mounted using 50% glycerol buffered in PBS and were stored in the dark at 4°C. Sections were examined with an observer ZI fluorescent microscope (Carl Zeiss, Thornwood, NY, USA) using appropriate filters (Chroma Technology, Bellow Falls, VT, USA).

### Flow Activated Cell Analyses

Nine mice in each experimental and control groups were used in this part of our study. Either the left or right MCC were used for the flow activated cell analyses. Immediately after euthanization the MCC was dissected under a dissection microscope and only the MCC was collected. Cells from the MCC were harvested by digestion with collagenase D (4mg/ml) (Roche Diagnostics, Mannheim, Germany) and dispase (4mg/ml) (Gibco, Grand Island, NY). Single cell suspensions were prepared by re-suspending cell pellets in 2 ml of fixed staining medium (HBSS+10mML of HEPES + 2%FBS) and passing through an 18-gauge needle followed by filtration through a 70μm strainer.

Single cell suspensions were analyzed on an LSRII flow cytometer (BD Biosciences, San Jose, California) and analyzed using BD FACS Diva analysis software (BD Biosciences). Gates for single cells and debris exclusion were made based on light scatter properties. For each sample, a minimum of 50,000 events was collected. Green fluorescent protein was excited using a 50mW 488nM laser and fluorescence detected from 505-550nM. Cyan fluorescent protein was excited using a 100mW 405nM laser and fluorescence detected from 425-475nM. mCherry was excited using a 100mW 561nm laser and fluorescence detected from 600-620nM.

### Micro-CT

Mineralized cartilage and subchondral bone was analyzed using micro-computerized tomography (SCANCO Medical AG, Brüttisellen, Switzerland). The samples were scanned in liquid, one at a time, with high resolution in a 16mm holder. Serial tomographic projections were acquired at 55kV and 145μA, with a voxel size of 6μm and 1000 projections per rotation collected at 300000μs. The DICOM images were transferred, segmented and reconstructed using the mimics software (Materialise, Belgium). In order to distinguish calcified tissue from non-calcified tissue, an automated algorithm using local threshold segmented the reconstructed grey scale images. Bone mineral density (BMD (mg/cc)), bone volume fraction (BVF (%)), trabecular thickness (Tb.Th (um)), and trabecular spacing (Tb.Sp (um)) were determined.

### Morphological Measurement

Radiographs of the mandibles were taken with a MX20 Radiography System (Faxitron X-Ray LLC, Lincolnshire, IL, USA) at a 26Kv for 5 seconds. We performed morphometric measurements on x-rays of mice subjected to altered loading and compared it with control triple transgenic mice. The parameters measured and compared were: 1) mandibular length (condylion to incisor process); 2) Condyle head length (the perpendicular distance from condylion to a line traced from the sigmoid notch to the deepest point in the concavity of the mandibular ramus) and; 3) Condyle head width (distance from the most anterior to the most posterior point of the condylar articular surface). Measurements were made using Digimizer^®^ Image software (MedCalc Software, Mariakerke, Belgium). Each measurement was done in triplicate and then the average was calculated.

### Histological Staining

Our histological sections were stained following a previously described protocol [19]. The 5μm- 7μm MCC sections remain adherent to glass slides through all of the process of staining and imaging. The first step was to image for GFP signals Dkk3 (green), Col2a1 (blue) and Col10a1 (red) and bone labels alizarin complexone (red) and calcein (green). Baseline imaging of the sections was performed with the observer ZI fluorescent microscope (Carl Zeiss, Thornwood, NY, USA) using a yellow fluorescent protein filter (eYFP, Chroma Cat 49003ET, EX: 500/20, EM: 535/30), a cyan fluorescent protein filter (CFP, Chroma Cat 49001ET, EX: 436/20, EM: 480/40), and a RFPcherry filter that was also used for detecting alizarin complexone staining (mCherry, Chroma Cat 49009ET, EX: 560/40, EM: 630/75). In the next step, the coverslip was removed by soaking in PBS and the sections from both the altered loading group and unloaded group were stained for EdU (Life Technologies, Grand Island, NY) and imaged. Subsequently, sections were stained for Tartrate Resistant Acid Phosphatase (TRAP) using ELF97 (Life Tech, E6589), which generates a yellow fluorescent signal. After imaging for TRAP, the coverslip was removed and the same slide was stained for Alkaline Phosphatase (AP) activity using a fluorescent fast red substrate (Sigma, #F8764-5G) and DAPI (Mol Probes #D-1306) and re-imaged. Finally the slide was rinsed in distilled water, stained with toluidine blue, and reimaged.

### RNA extraction and real-time PCR

Nine mice (9 condyles) in each experimental and control groups were used in this part of our study. Either the left or right MCC were used for the RNA extraction and gene expression analyses. The MCC along with the subchondral bone was minced and total RNA was extracted with TRIzol reagent (Invitrogen Life Technologies, CA, USA) using manufacturer’s protocol and as described previously[16]. The total RNA obtained was converted to cDNA using the ABI High Capacity cDNA Archive Kit (Applied Biosystems, Foster City, CA) following manufacturer’s protocol. Real-time PCR was performed for the expression of different genes in separate wells (singleplex assay) of 96-well plates in a reaction volume of 20μl. The relative expression in a test sample compared to a reference calibrator sample (^^Ct method) was used for the data analysis. The primers for target genes were purchased from the Applied Biosystem. Gene expression analyses were performed for Bone Morphogenic Protein 2 (*BMP2*)[20], SRY-box containing gene 9 (*Sox9*)[21], Indian Hedgehog (*Ihh*) [20], Collagen type 2 (*Col2a1*)[21], Collagen type 10 (*Col10a1*)[21], Sclerostin (*Sost*)[22] and Glyceraldehyde-3-phosphate dehydrogenase (GAPDH) as the internal control. The data were collected from three independent pooled samples (*n* = 9).

### Image Quantification

Cell proliferation in the MCC was quantified by measuring the EdU and DAPI positive pixels in the superficial and proliferative zone of the MCC and then calculating the percentage of EdU positive pixels over DAPI positive pixels. We examined TRAP activity in the MCC and subchondral bone by counting the number of yellow pixels (generated by ELF97) and dividing it by the total number of pixels in the subchondral bone region. Alkaline Phosphatase (AP) staining was quantified by measuring the distance from the most superficial cellular layer of MCC to the AP stain. Finally, sections were stained with Toluidine Blue (TB) to evaluate proteoglycan secretion within the MCC. TB stained area and TB distance mapping (in five different locations in the MCC) were also evaluated using adobe Photoshop (San Jose, CA)

### Statistical Analysis

Descriptive statistics were used to examine the distribution of bone volume fraction, tissue density, trabecular thickness, trabecular spacing, morphometric measurements (mandibular length, condylar length, condylar width), EdU positive cells, histomorphometric image analyses and gene expression analyses. A one Sample Kolmogorov-Smirnov test was used to examine the normality of data distribution. Outcomes were compared between the loaded and the unloaded group. Statistically significant differences among means were determined by unpaired t-test, with post hoc analysis by Mann-Whitney U test. All statistical tests were two sided and a p-value of <0.05 was deemed to be statistically significant. Statistical analyses were computed using Graph Pad Prism (San Diego, CA, USA)

## Results

### Altered Loading Causes Increase in chondrocytes proliferation and differentiation/maturation

**Fig 3:**
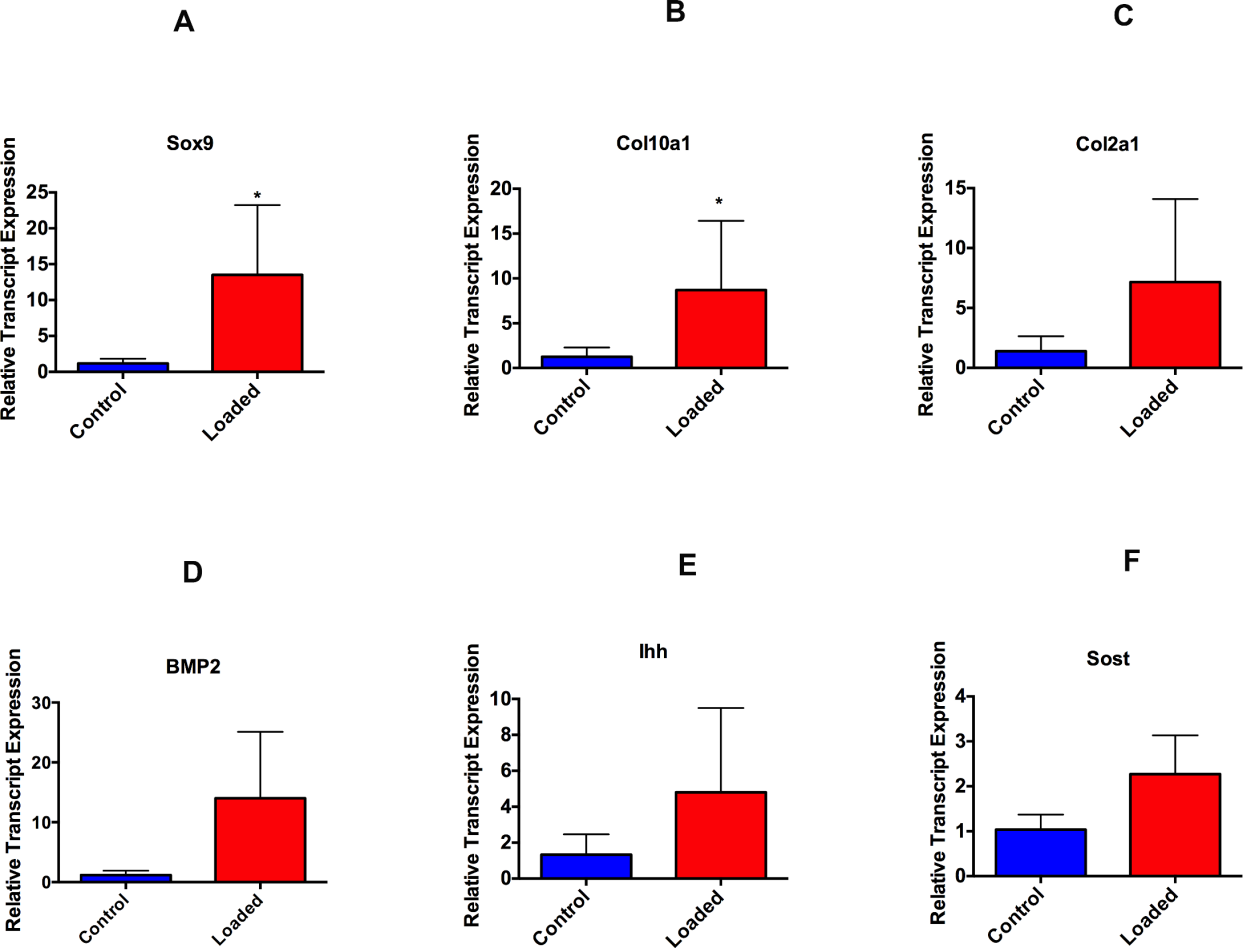
Altered loading leads to increase in proliferation (Sox9) and differentiation markers (Col10a1 and BMP2). 4-weeks-old male mice were exposed to altered and normal loading. Real time PCR analysis was performed for SRY-box containing gene 9(Sox9), Collagen 2 (Col2a1), Collagen X (Col10a1), Bone Morphogenic Protein 2 (BMP2), Indian Hedgehog (Ihh) and Sclerostin (SOST). Significant increase (p<0.05) were seen for Sox9 and Col10a1 expression. Histograms represent means ± SD.

Our histology showed that Dkk3-positive green cells are present in the superficial layer of fibrocartilaginous zone of MCC (Fig 2A). The Col2a1-positive blue and Col10a1-positive red cells are present in the pre-hypertrophic and hypertrophic zone of the MCC, respectively (Fig 2A). Furthermore, Col10a1 cells were present at the tidemark and in the mineralized region of the cartilage. The alizarin complexone and calcein bone labels were not distinct in the MCC, but were completely separated from each other in subchondral bone (Fig 2A). The flow analysis revealed that there was a significant increase in the number of Col2a1-positive (p<0.05) and Col10a1-positive (p<0.05) expressing cells with altered loading (Fig 2B). However, our gene expression, analysis only showed significantly increased expression of Col10a1 (p<0.05) and Sox9 (p<0.05) in the loading group when compared to the control group (Fig 3). Furthermore, there was a decrease in the number of Dkk3-positive expressing cells with altered loading, but was not significantly different from the control group (Fig 2B). Furthermore there was increase in the number of EdU positive cells in the loaded group (65.5% increase) signifies increase in proliferation (Fig 2C, 2D and 2E).

### Altered Compressive Loading Causes Increase in Bone Volume and Trabecular Thickness

**Fig 4:**
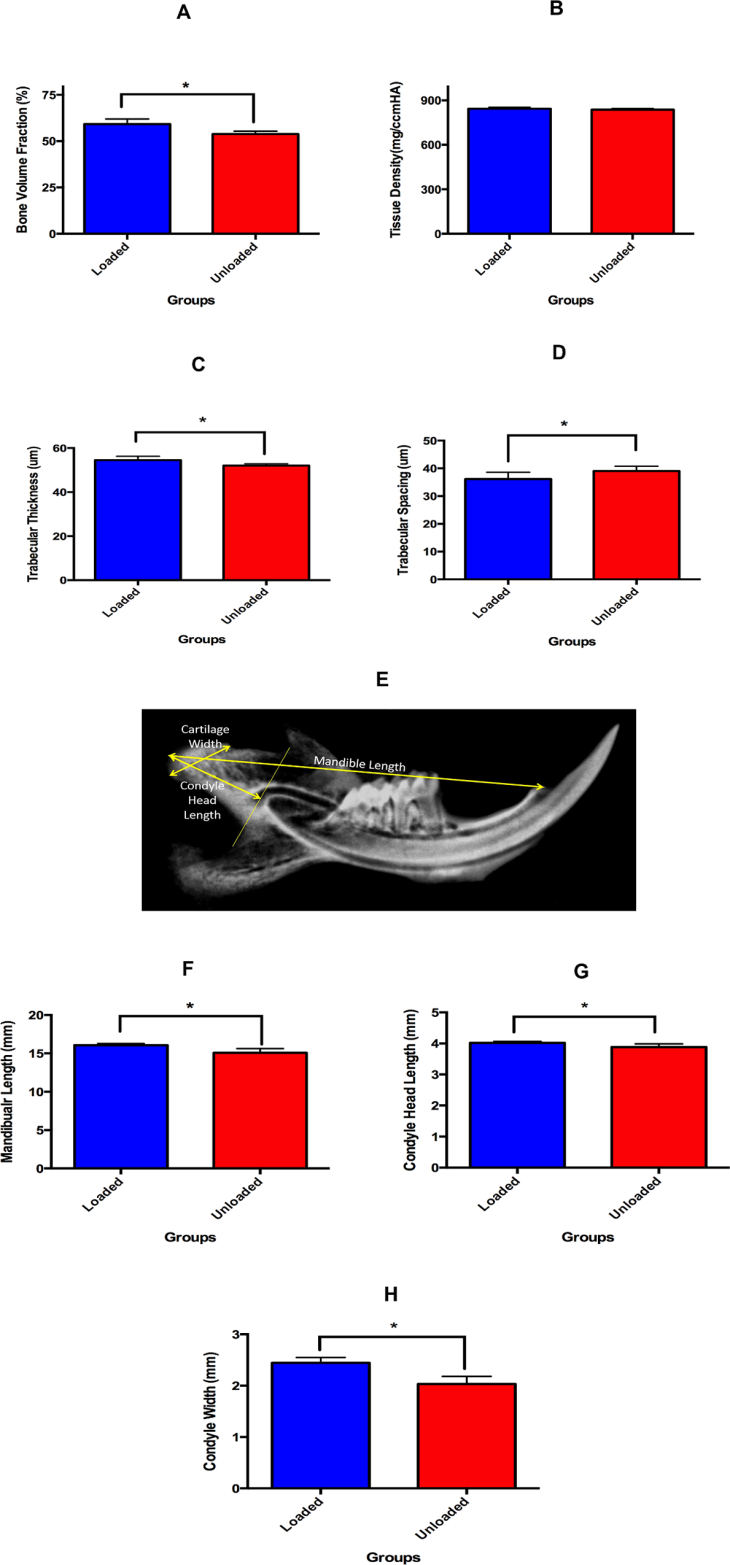
Altered loading of MCC increases the bone volume and trabecular thickness. Micro-CT and morphometric measurements of altered compressive loading and unloaded groups. (4A) micro-CT analysis of bone volume fraction, (4B) micro-CT analysis of trabecular thickness and, (4C) micro-CT analysis of trabecular spacing, (4D) Representative image of the morphometric parameters measured in the experimental and control groups, (4E) Mandibular length on the 2D x-ray (faxitron), (4F) Condylar head length on the 2D x-ray (faxitron) and, (4G) Condyle width on the 2D x-ray (faxitron). (*: p<0.05 = significant difference between the altered loading and control group. Histograms represent means ± SD.

The *ex-vivo* micro-CT protocol provided sufficient spatial resolution for quantitative comparison of the structure and morphology of the mineralized cartilage and subchondral bone between the experimental and the control group. The analysis revealed a significant increase in the bone volume fraction (10.56%) (p<0.05) with altered loading (Fig 4A). This was paralleled by an increase in trabecular thickness (p<0.05) and decrease in trabecular spacing (p<0.05) in the loaded group when compared to the control group (Fig 4B and 4C). The trabecular thickness increased by 6.07% and there was a 9.02% decrease in the trabecular spacing in the altered loading group.

### Altered Loading Causes Increase in Mandibular Length, Condyle Head Length and Cartilage Width

Altered compressive loading of the TMJ did cause an increase in morphometric parameters after 5 days of loading. The mandibular length in the loaded group (16.08 ± 0.21) was significantly greater (p<0.05) than the unloaded group (15.08 ± 0.53) (Fig 4D and 4E). There was a 6.67% increase in the length of the mandible with the 5 days of altered loading. Similarly, condyle head length was significantly greater (p<0.05) in the loaded group (4.01 ± 0.04) when compared to unloaded group (3.88 ± 0.09) (Fig 4D and 4F). Moreover, the condyle width was also significantly more (p<0.05) in the loaded group (2.44 ± 0.10) then unloaded group (2.03 ± 0.15) (Fig 4D and 4G). The condyle head length and condyle width increased by 5.15% and 20.19% in the loaded group when compared to the unloaded group.

### Altered Compressive Loading Causes Increase in Mineral apposition

**Fig 5:**
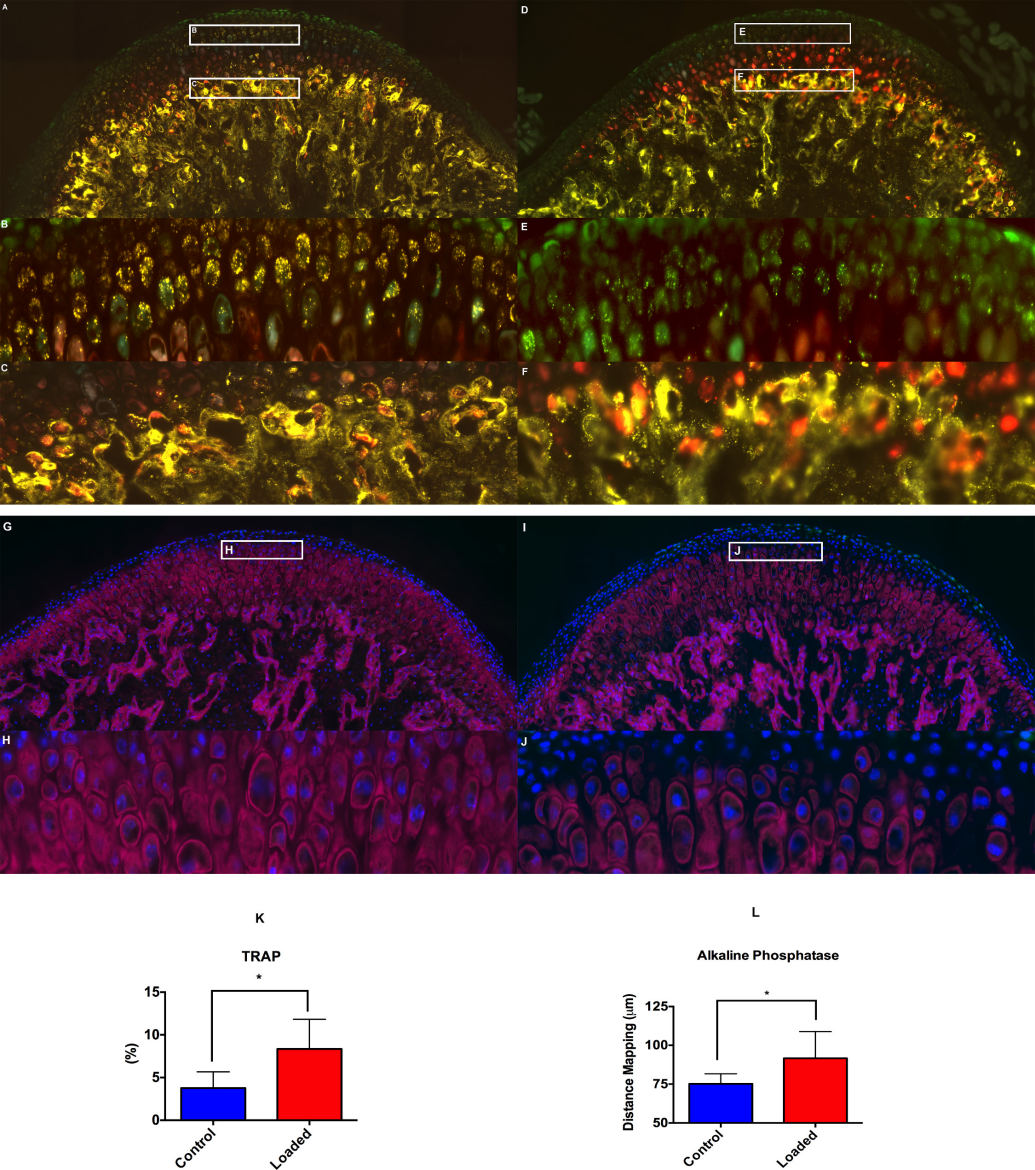
Altered loading increases TRAP activity and mineralization. The histological changes (TRAP & AP) within the mandibular condylar cartilage in the altered loaded and control groups. Fig 5A–5C shows staining for TRAP in the altered loaded group, (5A) TRAP stained sagittal section of the MCC along with the subchondral bone, (5B) TRAP positive cells (punctate appearance) cells in the proliferative zone of the MCC. Number and the intensity of the TRAP positive cells in the proliferative zone of MCC are more in the loaded group, when compared to the control group, (5C) TRAP positive cells in the subchondral bone of the loaded group. Fig 5D–5F show staining for TRAP positive cells in control group, (5D) TRAP stained sagittal section of the MCC along with the subchondral bone in the control group, (5E) TRAP positive cells (punctate appearance) cells in the proliferative zone of the MCC, (5F) TRAP positive cells in the subchondral bone of the control group. (5G-5H) AP staining in the altered loaded group, (5G) AP staining in MCC and subchondral bone of loaded group, (5H) AP staining in the proliferative and fibrocartilaginous zone of the MCC. (5I-5J) AP staining in the control group, (5I) AP staining in the MCC and subchondral bone of the control group, (5J) AP staining in the proliferative zone of MCC but not in the fibrocartilaginous region of MCC. (5K) Quantification of TRAP positive pixels, (5L) Quantification of distance map. Histograms represent means ± SD. * Statistically significant difference between the altered loaded side and the control group. Scale bar = 500μm.

**Fig 6:**
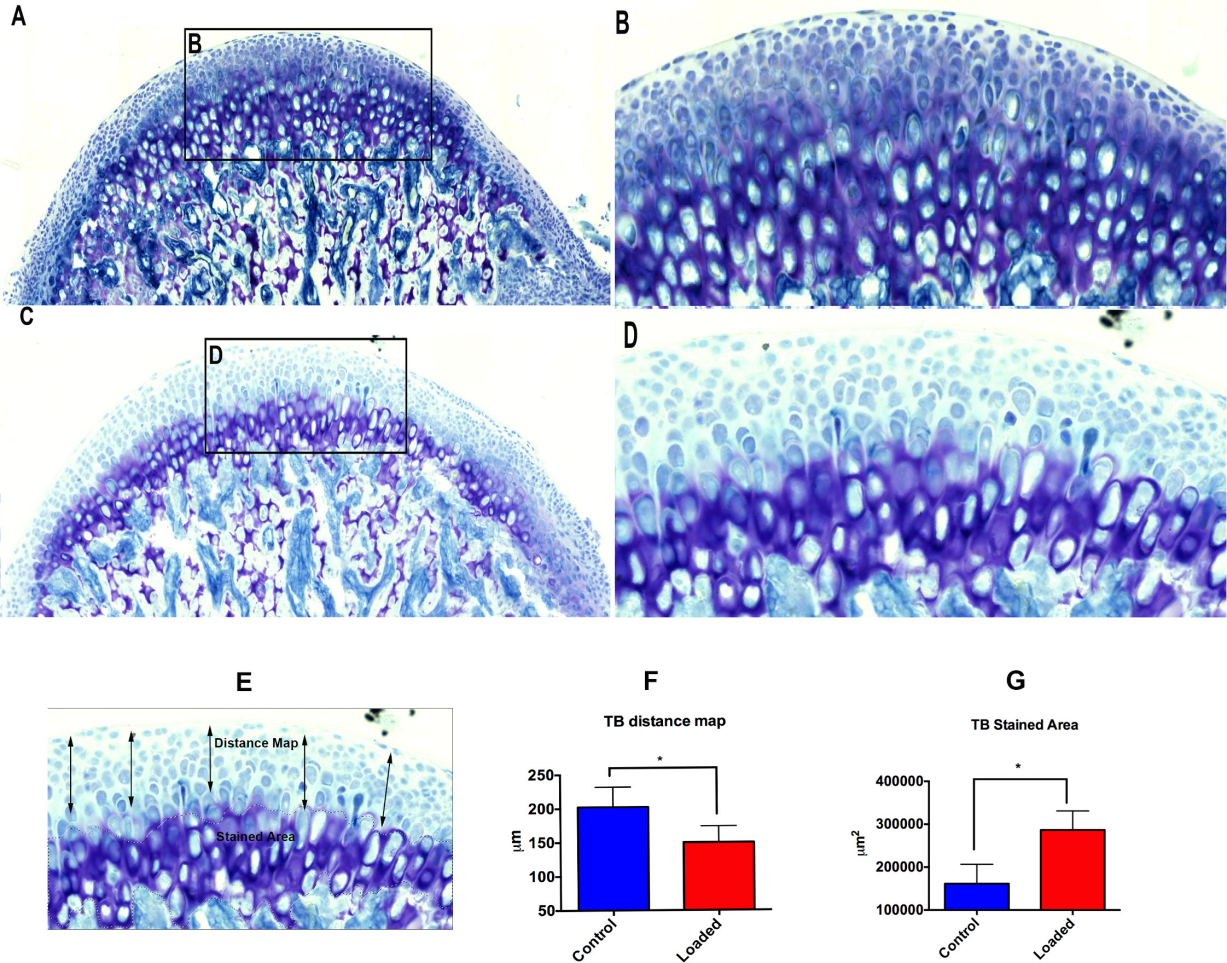
Altered loading increases proteoglycan accumulation. The histological changes (TB Staining) within the mandibular condylar cartilage in the altered compressive loaded and control groups. Fig 6A–6B shows TB staining in the altered loaded group. Strong staining for proteoglycans is observed. The thickness of the mineralized cartilage is more. (6A) TB stained sagittal section of the MCC along with the subchondral bone, (6B) Intense proteoglycan staining in the fibrocartilaginous zone of the MCC, (6C-6D) TB staining in the altered unloaded group shows weaker staining when compared to the loaded group and less thickness of the mineralized cartilage. (6C) TB staining of the sagittal section of the MCC along with the subchondral bone in the unloaded group, (6D) thickness of mineralized fibrocartilage is less. (6F) Quantification of TB distance map, (6G) Quantification of TB stained area. Histograms represent means ± SD. * Statistically significant difference between the altered loaded side and the control group. Scale bar = 500μm.

The altered compressive loading resulted in increased mineralized matrix apposition and increase in calcified cartilage, which was evident by TRAP (Fig 5A, 5B and 5K), alkaline phosphatase (Fig 5G, 5H and 5L) and toluidine blue staining (Fig 6A, 6B, 6F and 6G). In the altered loading (Fig 5A and 5B) and control group (Fig 5D and 5E) the TRAP positive cells were present in the proliferative zone of the MCC. However, the TRAP positive cells were much more in the loaded group (Fig 5B), when compared to control group (Fig 5E). We believe that these TRAP positive cells were not osteoclasts/macrophage specific, because the TRAP in the cartilage has a punctate intracellular distribution within a cell in contrast to the homogeneous distribution of TRAP within multinucleated osteoclastic cells in the subchondral bone. However, we were not able to detect any differences in the TRAP staining of the subchondral bone between the loaded (Fig 5C) and the Control group (Fig 5F).

Similarly, the AP stain was present in the proliferative zone and more towards the fibrocartilage layer in the loaded group (Fig 5G and 5H), when compared to the unloaded group (Fig 5I and 5J). This finding was further confirmed by the increase in Toluidine blue staining throughout the mineralized region of the fibrocartilage in the altered compressive loading group (Fig 6A and 6B).

## Discussion

Mandibular condylar cartilage is affected by mechanical environment and it has been speculated that under stimulation and over stimulation can induce degenerative changes[23]. Adaptive remodeling of MCC is necessary for the homeostasis and proper functioning of the TMJ. In this study we have used the forced mouth-opening model to alter the mechanical environment of MCC of the TMJ. The altered loading in our study has resulted in specific and consistent differences in the MCC and subchondral bone. We used triple transgenic mice (Dkk3 X Col2a1 X Col10a1) as we wanted to study the cellular effects of altered loading on fibrocartilage and mature cartilage. Dkk3 is primarily expressed in fibrocartilaginous zone of the MCC while Col2a1 and Col10a1 are expressed in the pre-hypertrophic and hypertrophic zone of mature cartilage. We believe that the use of the triple colored transgenic mice and the histological features can contribute to an improved understanding of the molecular and cellular sequence of events after altered loading. The mandible is considered to be a class III lever; the condyle acts as a fulcrum, the masticatory muscles as applied force and bite pressure as a resistance[24–27]. Historically, loading of the TMJ is understood to be compressive in nature. i.e. the force applied at the loading surfaces of the MCC is compressive (Fig 1). Experimental and computer modeling studies have indicated that MCC is subjected to both compressive and tensile forces, and the forces vary significantly among different species and therefore is difficult to explain the precise forces on the MCC when the mechanical environment is altered[28–32]. In our study we have chosen 0.5N of force, as Sobue et al. have showed that force less than 0.5N has less anabolic effect on the MCC[5]. We applied the static force only for 5 days, as Lai et al. have showed that shorter duration of static compression (1hour each day for 14 days) on growth plate are anabolic[33].

Mandibular condylar cartilage has a heterogeneous cell population and consists of fibroblast like cells, fibrochondrocytes and chondrocytes[34]. The nature and behavior of these cells remains poorly understood during altered loading. The MCC plays a major role in absorbing the mechanical forces exerted by various oral functions and para-functions. It has been shown that loading of the MCC within the adaptive capacity may stimulate remodeling and increased synthesis of the extracellular matrix. Our flow activated cell analysis showed an increased number of Col2a1blue cells (30% increased when compared to control) and Col10a1red cells (100% increased when compared to control) with altered loading of MCC. Numerous in vitro studies in MCC and articular cartilage have shown an increase in cell differentiation and increased proteoglycan synthesis after mechanically loading the articular chondrocytes [35–39]. Similarly, Yang et al. in their *in vitro* study with articular chondrocytes showed a two-fold increase in type X collagen with mechanical loading[40]. Moreover, Sobue et al. showed an increase in Col2a1 and Col10a1 with 0.5N of compressive load on the MCC in mice [5]. Our experiments show similar results and it may be due to accelerated maturation/lineage progression with altered compressive load. Furthermore, we show a decrease in the number of Dkk3-positive (green) cells with loading and it may be due to finite number of Dkk3 cells present in the fibrocartilaginous zone of the MCC, and which may progress to type 1 collagen expressing cells thus accelerating the differentiation/lineage progression (data not shown). Dkk3 expression has been upregulated in osteoarthritis, suggesting its role in the pathogenesis of the diseases.

The altered compressive loading in the subchondral bone caused increased mineralization, increased bone volume fraction, increased trabecular thickness and decreased trabecular spacing. It has been postulated that subchondral bone adapts to applied load/stress. A sequential and coordinated response of modeling and remodeling control this adaptive response. Bone formation is hypothesized to be driven by the magnitude, rate, and duration of applied bone strain[41]. Our result shows an increase in bone volume and trabecular thickness with the static 0.5N load and a plausible reason could be that the strain felt by the subchondral bone cells was above the maximum strain threshold, thus leading to bone formation. The mechanism by which bone strain induces bone formation is unknown, however, microcrack propagation through the bone matrix has been shown to stimulate bone remodeling[42, 43]. Our results are contrary to Sobue et al. as they showed a decrease in bone volume and trabecular thickness with loading. These differences could possibly be explained by different strains of mice, as well as different gender and age [5].

Our morphometric measurement showed increased mandibular length, increased condylar length and increased mandibular condyle width. These increases in morphometric parameters with altered loading can be due to increased proliferation of chondrocytes and increased secretion of pericellular and extracellular matrix. Sobue et al. have shown increased proliferation with 0.5N of static compressive force on the MCC[5]. Similarly, Pirttiniemi et al. and Chen et al. showed that reduced loading leads to a lower number of proliferating chondrocytes and a thinner cartilaginous layer[4, 44, 45]. Our data shows that static compressive forces can be used to modify the growth of the mandible, however, whether the effects of the static force on the MCC are transitory, needs to be further evaluated.

Our histology shows increased mineralized matrix (toluidine blue & alkaline phosphatase staining) with altered loading, when compared to unloaded group. In vitro studies have demonstrated that static compressive loading leads to increased proteoglycan synthesis[46, 47]. Conversely, decreased loading of the MCC of the joint results in decreased proteoglycan synthesis[48]. In the MCC the toluidine blue staining is found in the pre-hypertrophic and hypertrophic zone. With altered loading the toluidine blue stain approaches the articular surface of the MCC, where as in the unloaded group it was in the pre-hypertrophic and hypertrophic zones of MCC. Concomitant with the increase in toluidine blue staining (proteoglycan accumulation), there was increase in the number of Col10a1 cells and Col2a1 cells.

We were surprised to see the TRAP activity in the cells within the uncalcified fibrocartilage. The TRAP activity was primarily in the proliferative zone of MCC and was more intense in the loaded group, when compared to the unloaded group. The punctate appearance of TRAP has been observed previously in the histology of cartilage and macrophages[49]. However, the role of TRAP in remodeling of the fibrocartilage prior to mineralization is still not clear. Similarly, we noted increased in AP staining in the altered compressive loading group. The AP activity in the altered loading group was synonymous with our micro-CT results, which showed increase in bone volume fraction in the loaded group when compared to unloaded group.

Limitations and future directions: Our study is the first study using combination of strategies to study the effect of altered loading on MCC and subchondral bone. The limitation of this study was our inability to accurately define the force felt by the MCC. However, TMJ is a complex joint and scientific literature lacks the force range, which could stimulate adaptive remodeling of the MCC. Another limitation of our study could be use of male mice as TMJ disorders are more prevalent in female mice, but numerous studies have been done in the past on female mice and we wanted to study whether the mandibular growth could be modified using altered loading model. Furthermore, there are equal numbers of male and female patients with small mandible. Our future studies are focusing on applying the altered load in an adult animal model and sacrificing the animals at different time points to study the concept of adaptive remodeling.

## Conclusions

In conclusion altered loading of the MCC leads to:

1. Increased cellular proliferation (number of EDU positive cells) and differentiation.
2. Increased mandibular length, condylar head length and condylar width.
3. Increased subchondral bone volume, increased trabecular thickness and decreased trabecular spacing.
4. Increased mineralization as evident by the TRAP, alkaline phosphatase and toluidine blue staining

## References

[1] Beek M, Koolstra JH, van Ruijven LJ, van Eijden TM. Three-dimensional finite element analysis of the human temporomandibular joint disc. Journal of biomechanics. 2000;33(3):307–16. . 10673114 10.1016/s0021-9290(99)00168-2

[2] Hu K, Qiguo R, Fang J, Mao JJ. Effects of condylar fibrocartilage on the biomechanical loading of the human temporomandibular joint in a three-dimensional, nonlinear finite element model. Medical engineering & physics. 2003;25(2):107–13. . 12538065 10.1016/s1350-4533(02)00191-1

[3] Boyd RL, Gibbs CH, Mahan PE, Richmond AF, Laskin JL. Temporomandibular joint forces measured at the condyle of Macaca arctoides. American journal of orthodontics and dentofacial orthopedics: official publication of the American Association of Orthodontists, its constituent societies, and the American Board of Orthodontics. 1990;97(6):472–9. 10.1016/S0889-5406(05)80027-7 . 2353676

[4] Chen J, Sorensen KP, Gupta T, Kilts T, Young M, Wadhwa S. Altered functional loading causes differential effects in the subchondral bone and condylar cartilage in the temporomandibular joint from young mice. Osteoarthritis Cartilage. 2009;17(3):354–61. 10.1016/j.joca.2008.05.021 18789726 PMC2646810

[5] Sobue T, Yeh WC, Chhibber A, Utreja A, Diaz-Doran V, Adams D, et al Murine TMJ loading causes increased proliferation and chondrocyte maturation. J Dent Res. 2011;90(4):512–6. 10.1177/0022034510390810 21248355 PMC3065547

[6] Luder HU, Leblond CP, von der Mark K. Cellular stages in cartilage formation as revealed by morphometry, radioautography and type II collagen immunostaining of the mandibular condyle from weanling rats. Am J Anat. 1988;182(3):197–214. 10.1002/aja.1001820302 . 3213819

[7] Mizoguchi I, Takahashi I, Nakamura M, Sasano Y, Sato S, Kagayama M, et al An immunohistochemical study of regional differences in the distribution of type I and type II collagens in rat mandibular condylar cartilage. Archives of oral biology. 1996;41(8–9):863–9. . 9022924 10.1016/s0003-9969(96)00021-0

[8] Kuroda S, Tanimoto K, Izawa T, Fujihara S, Koolstra JH, Tanaka E. Biomechanical and biochemical characteristics of the mandibular condylar cartilage. Osteoarthritis and cartilage / OARS, Osteoarthritis Research Society. 2009;17(11):1408–15. 10.1016/j.joca.2009.04.025 . 19477310

[9] Tanaka E, Yamano E, Dalla-Bona DA, Watanabe M, Inubushi T, Shirakura M, et al Dynamic compressive properties of the mandibular condylar cartilage. Journal of dental research. 2006;85(6):571–5. . 16723658 10.1177/154405910608500618

[10] Singh M, Detamore MS. Tensile properties of the mandibular condylar cartilage. Journal of biomechanical engineering. 2008;130(1):011009 10.1115/1.2838062 . 18298185

[11] Glinka A, Wu W, Delius H, Monaghan AP, Blumenstock C, Niehrs C. Dickkopf-1 is a member of a new family of secreted proteins and functions in head induction. Nature. 1998;391(6665):357–62. 10.1038/34848 . 9450748

[12] Krupnik VE, Sharp JD, Jiang C, Robison K, Chickering TW, Amaravadi L, et al Functional and structural diversity of the human Dickkopf gene family. Gene. 1999;238(2):301–13. . 10570958 10.1016/s0378-1119(99)00365-0

[13] Luke GN, Castro LF, McLay K, Bird C, Coulson A, Holland PW. Dispersal of NK homeobox gene clusters in amphioxus and humans. Proc Natl Acad Sci U S A. 2003;100(9):5292–5. 10.1073/pnas.0836141100 12704239 PMC154338

[14] Meng J, Ma X, Ma D, Xu C. Microarray analysis of differential gene expression in temporomandibular joint condylar cartilage after experimentally induced osteoarthritis. Osteoarthritis Cartilage. 2005;13(12):1115–25. 10.1016/j.joca.2005.03.010 . 15905105

[15] Maye P, Fu Y, Butler DL, Chokalingam K, Liu Y, Floret J, et al Generation and characterization of Col10a1-mcherry reporter mice. Genesis. 2011;49(5):410–8. 10.1002/dvg.20733 . 21328521 PMC5638041

[16] Chen J, Utreja A, Kalajzic Z, Sobue T, Rowe D, Wadhwa S. Isolation and characterization of murine mandibular condylar cartilage cell populations. Cells Tissues Organs. 2012;195(3):232–43. 10.1159/000325148 21646777 PMC3388270

[17] Ahmad M, Hollender L, Anderson Q, Kartha K, Ohrbach R, Truelove EL, et al Research diagnostic criteria for temporomandibular disorders (RDC/TMD): development of image analysis criteria and examiner reliability for image analysis. Oral Surg Oral Med Oral Pathol Oral Radiol Endod. 2009;107(6):844–60. 10.1016/j.tripleo.2009.02.023 19464658 PMC3139469

[18] Jiang X, Kalajzic Z, Maye P, Braut A, Bellizzi J, Mina M, et al Histological analysis of GFP expression in murine bone. The journal of histochemistry and cytochemistry: official journal of the Histochemistry Society. 2005;53(5):593–602. 10.1369/jhc.4A6401.2005 . 15872052

[19] Dyment NA, Hagiwara Y, Jiang X, Huang J, Adams DJ, Rowe DW. Response of knee fibrocartilage to joint destabilization. Osteoarthritis Cartilage. 2015;23(6):996–1006. 10.1016/j.joca.2015.01.017 . 25680653 PMC4757847

[20] Seki K, Hata A. Indian hedgehog gene is a target of the bone morphogenetic protein signaling pathway. J Biol Chem. 2004;279(18):18544–9. 10.1074/jbc.M311592200 . 14981086

[21] Matsubara T, Kida K, Yamaguchi A, Hata K, Ichida F, Meguro H, et al BMP2 regulates Osterix through Msx2 and Runx2 during osteoblast differentiation. J Biol Chem. 2008;283(43):29119–25. 10.1074/jbc.M801774200 18703512 PMC2662012

[22] van Bezooijen RL, Roelen BA, Visser A, van der Wee-Pals L, de Wilt E, Karperien M, et al Sclerostin is an osteocyte-expressed negative regulator of bone formation, but not a classical BMP antagonist. The Journal of experimental medicine. 2004;199(6):805–14. 10.1084/jem.20031454 15024046 PMC2212719

[23] Utreja A, Dyment NA, Yadav S, Villa MM, Li Y, Jiang X, et al Cell and matrix response of temporomandibular cartilage to mechanical loading. Osteoarthritis Cartilage. 2015 10.1016/j.joca.2015.08.010 . 26362410 PMC4757844

[24] Herring SW, Liu ZJ. Loading of the temporomandibular joint: anatomical and in vivo evidence from the bones. Cells Tissues Organs. 2001;169(3):193–200. 47882. . 11455114 10.1159/000047882

[25] Liu ZJ, Herring SW. Bone surface strains and internal bony pressures at the jaw joint of the miniature pig during masticatory muscle contraction. Arch Oral Biol. 2000;45(2):95–112. . 10716614 10.1016/s0003-9969(99)00127-2

[26] Hylander WL. The human mandible: lever or link? Am J Phys Anthropol. 1975;43(2):227–42. 10.1002/ajpa.1330430209 . 1101706

[27] Smith RJ. Mandibular biomechanics and temporomandibular joint function in primates. Am J Phys Anthropol. 1978;49(3):341–9. 10.1002/ajpa.1330490307 . 103437

[28] Huddleston Slater JJ, Visscher CM, Lobbezoo F, Naeije M. The intra-articular distance within the TMJ during free and loaded closing movements. J Dent Res. 1999;78(12):1815–20. . 10598911 10.1177/00220345990780120801

[29] Henderson SE, Lowe JR, Tudares MA, Gold MS, Almarza AJ. Temporomandibular joint fibrocartilage degeneration from unilateral dental splints. Arch Oral Biol. 2015;60(1):1–11. 10.1016/j.archoralbio.2014.08.022 25247778 PMC4252753

[30] Herring SW. TMJ anatomy and animal models. J Musculoskelet Neuronal Interact. 2003;3(4):391–4; discussion 406–7. 15758330 PMC2821032

[31] Korioth TW, Hannam AG. Mandibular forces during simulated tooth clenching. Journal of orofacial pain. 1994;8(2):178–89. . 7920353

[32] Hylander WL. Experimental analysis of temporomandibular joint reaction force in macaques. Am J Phys Anthropol. 1979;51(3):433–56. 10.1002/ajpa.1330510317 . 532828

[33] Lai A, Chow DH, Siu SW, Leung SS, Lau EF, Tang FH, et al Effects of static compression with different loading magnitudes and durations on the intervertebral disc: an in vivo rat-tail study. Spine (Phila Pa 1976). 2008;33(25):2721–7. 10.1097/BRS.0b013e318180e688 . 19050577

[34] Kantomaa T, Tuominen M, Pirttiniemi P. Effect of mechanical forces on chondrocyte maturation and differentiation in the mandibular condyle of the rat. J Dent Res. 1994;73(6):1150–6. . 8046103 10.1177/00220345940730060401

[35] Lane Smith R, Trindade MC, Ikenoue T, Mohtai M, Das P, Carter DR, et al Effects of shear stress on articular chondrocyte metabolism. Biorheology. 2000;37(1–2):95–107. . 10912182

[36] Lee DA, Bader DL. Compressive strains at physiological frequencies influence the metabolism of chondrocytes seeded in agarose. Journal of orthopaedic research: official publication of the Orthopaedic Research Society. 1997;15(2):181–8. 10.1002/jor.1100150205 . 9167619

[37] Shelton JC, Bader DL, Lee DA. Mechanical conditioning influences the metabolic response of cell-seeded constructs. Cells, tissues, organs. 2003;175(3):140–50. 74630. . 14663157 10.1159/000074630

[38] Copray JC, Jansen HW, Duterloo HS. Effects of compressive forces on proliferation and matrix synthesis in mandibular condylar cartilage of the rat in vitro. Archives of oral biology. 1985;30(4):299–304. . 3857899 10.1016/0003-9969(85)90001-9

[39] Copray JC, Jansen HW, Duterloo HS. An in-vitro system for studying the effect of variable compressive forces on the mandibular condylar cartilage of the rat. Archives of oral biology. 1985;30(4):305–11. . 3857900 10.1016/0003-9969(85)90002-0

[40] Yang X, Vezeridis PS, Nicholas B, Crisco JJ, Moore DC, Chen Q. Differential expression of type X collagen in a mechanically active 3-D chondrocyte culture system: a quantitative study. Journal of orthopaedic surgery and research. 2006;1:15 10.1186/1749-799X-1-15 17150098 PMC1764003

[41] Xie L, Jacobson JM, Choi ES, Busa B, Donahue LR, Miller LM, et al Low-level mechanical vibrations can influence bone resorption and bone formation in the growing skeleton. Bone. 2006;39(5):1059–66. 10.1016/j.bone.2006.05.012 . 16824816

[42] Yuan XL, Meng HY, Wang YC, Peng J, Guo QY, Wang AY, et al Bone-cartilage interface crosstalk in osteoarthritis: potential pathways and future therapeutic strategies. Osteoarthritis and cartilage / OARS, Osteoarthritis Research Society. 2014;22(8):1077–89. 10.1016/j.joca.2014.05.023 . 24928319

[43] Wang X, Niebur GL. Microdamage propagation in trabecular bone due to changes in loading mode. Journal of biomechanics. 2006;39(5):781–90. 10.1016/j.jbiomech.2005.02.007 . 16488217

[44] Pirttiniemi P, Kantomaa T, Salo L, Tuominen M. Effect of reduced articular function on deposition of type I and type II collagens in the mandibular condylar cartilage of the rat. Archives of oral biology. 1996;41(1):127–31. . 8833602 10.1016/0003-9969(95)00102-6

[45] Pirttiniemi P, Kantomaa T, Sorsa T. Effect of decreased loading on the metabolic activity of the mandibular condylar cartilage in the rat. European journal of orthodontics. 2004;26(1):1–5. . 14994876 10.1093/ejo/26.1.1

[46] Carvalho RS, Yen EH, Suga DM. Glycosaminoglycan synthesis in the rat articular disk in response to mechanical stress. Am J Orthod Dentofacial Orthop. 1995;107(4):401–10. . 7709905 10.1016/s0889-5406(95)70093-5

[47] Okazaki J, Kamada A, Higuchi Y, Kanabayashi T, Sakaki T, Gonda Y. Age changes in the rat temporomandibular joint articular disc: a biochemical study on glycosaminoglycan content. J Oral Rehabil. 1996;23(8):536–40. . 8866266 10.1111/j.1365-2842.1996.tb00892.x

[48] Hinton RJ. Effect of altered masticatory function on [3H]-thymidine and [35S]-sulfate incorporation in the condylar cartilage of the rat. Acta Anat (Basel). 1988;131(2):136–9. . 3369280 10.1159/000146501

[49] Hayman AR, Bune AJ, Bradley JR, Rashbass J, Cox TM. Osteoclastic tartrate-resistant acid phosphatase (Acp 5): its localization to dendritic cells and diverse murine tissues. The journal of histochemistry and cytochemistry: official journal of the Histochemistry Society. 2000;48(2):219–28. . 10639488 10.1177/002215540004800207

